# Tubulation repair mitigates misdirection of regenerating motor axons across a sciatic nerve gap in rats

**DOI:** 10.1038/s41598-018-21652-y

**Published:** 2018-02-21

**Authors:** Dan Liu, Daguo Mi, Tuanjie Zhang, Yanping Zhang, Junying Yan, Yaxian Wang, Xuefeng Tan, Ying Yuan, Yumin Yang, Xiaosong Gu, Wen Hu

**Affiliations:** 1 Key Laboratory for Neuroregeneration of Ministry of Education and Co-innovation Center for Neuroregeneration of Jiangsu Province, Nantong University, Nantong, Jiangsu 226001 China; 2 The Affiliated Hospital of Nantong University, Nantong, Jiangsu 226001 China; 3 Department of Orthopedics, Nantong Hospital of Traditional Chinese Medicine, Nantong, Jiangsu 226001 China; 4 School of Medicine, Nantong University, Nantong, Jiangsu 226001 China; 5 Present Address: Department of Burns and Plastic Surgery and Cosmetology, Longyan First Hospital, Longyan, Fujian 364000 China

## Abstract

The repair of peripheral nerve laceration injury to obtain optimal function recovery remains a big challenge in the clinic. Misdirection of regenerating axons to inappropriate target, as a result of forced mismatch of endoneurial sheaths in the case of end-to-end nerve anastomosis or nerve autografting, represents one major drawback that limits nerve function recovery. Here we tested whether tubulation repair of a nerve defect could be beneficial in terms of nerve regeneration accuracy and nerve function. We employed sequential retrograde neuronal tracing to assess the accuracy of motor axon regeneration into the tibial nerve after sciatic nerve laceration and entubulation in adult Sprague-Dawley rats. In a separate cohort of rats with the same sciatic nerve injury/repair protocols, we evaluated nerve function recovery behaviorally and electrophysiologically. The results showed that tubulation repair of the lacerated sciatic nerve using a 3-6-mm-long bioabsorbable guidance conduit significantly reduced the misdirection of motor axons into the tibial nerve as compared to nerve autografting. In addition, tubulation repair ameliorated chronic flexion contracture. This study suggests that tubulation repair of a nerve laceration injury by utilizing a bioresorbable nerve guidance conduit represents a potential substitute for end-to-end epineurial suturing and nerve autografting.

## Introduction

Peripheral nerve injury represents the major cause of peripheral neuropathy, a lifelong morbidity which affects the quality of life and/or work ability due to numbness, pain, muscle weakness and cold intolerance^1–3^. Epidemiological evidence shows that the incidence of peripheral nerve injury is ~13.9/100,000 persons per year^4^; in the US alone, estimated 20 million people sustain peripheral neuropathy^5^. Notably, about 80% of all peripheral nerve injuries take place in the upper extremities or ~63% at the wrist level or in the hand^4^, substantially affecting hand function. Surgical repair of a broken nerve is indispensible for the recovery of its function; however, the functional recovery of a lacerated major nerve can hardly be complete in adults even with the most advanced microsurgical intervention^1^. From clinical point of view, the recovery of function after nerve injury is influenced by age of the patient, level of injury, type of nerve, mechanism of injury, timeliness of repair, and patient’s compliance to rehabilitative training^1^. Neurobiologically, nerve function recovery is jointly determined by neuronal survival, velocity of axonal regeneration, amount and accuracy of re-innervation, condition of the denervated target, and plasticity of the central nervous system after nerve injury^1^. Among all these aspects, regeneration accuracy of a major nerve remains a domain with inadequate knowledge, especially in terms of therapeutic strategy.

Mitigation of the misdirected regrowth of axons represents one of the most challenging tasks in the area of peripheral nerve regeneration. Spinal nerves, peripheral nerves that are vulnerable to traumatic injury^4^, comprise sensory, motor, and autonomic nerve fibers. Physiologically, nerve fibers of different nature follow predefined topographic distribution to precisely innervate the skin (sensory), skeletal muscles (motor), and vasculature, arrector pili muscles and subcutaneous glands (autonomic). In the case of nerve laceration injury, however, the mismatch between regenerating axons and the targets becomes unavoidable^6,7^, since it is impossible to guarantee precise endoneurium-to-endoneurium reconnection. The disruption of endoneurial alignment represents a big challenge for pathway finding of regenerating axons. Consequently, regenerated motor axons may misroute into inappropriate skeletal muscles or sensory targets, and sensory axons may erroneously re-innervate neuromuscular junctions^6^. Studies have shown that Schwann cells of the peripheral nerve express motor and sensory phenotypes that differentially regulate nerve regeneration^8^ and regenerating axons hold the potential to preferentially re-innervate appropriate targets^9^. In direct nerve anastomosis however, forced mismatch between endoneuria of the proximal nerve stump and endoneurial pathways of different nature in the distal stump prevents regenerating axons from potentially selective entry into appropriate endoneurium. The forced endoneurial mismatch occurs at the co-aptation^10^ and can be further complicated in the case of nerve autografting. Therefore, the search for therapeutic strategy to minimize mismatch of regenerating axons and pathways of axonal regrowth represents a significant attempt.

Intriguingly, randomized controlled clinical studies have shown that better functional outcome can be achieved by tubulation repair of nerve injury, i.e. intentionally leaving a 3–5 mm long gap between the two nerve stumps within a conduit, as compared to end-to-end nerve suturing^11,12^. Less cold intolerance was observed after the repair of median nerve injury in the forearm with non-degradable silicone tubes^11^, and sensory recovery was improved in digital nerves repaired by biodegradable polyglycolic acid tubes^12^, when compared to direct nerve anastomosis. In murine models, tubulation repair has been shown to reduce aberrant motor regeneration and promote function recovery when combined with a luminal nerve graft^13^, and tubulation with a bioabsorbable nerve counduit has been proposed to substitute end-to-end epineurial neurorrhaphy^14,15^. However, it remains to be understood whether and to what extent conduit repair can improve regeneration accuracy and how it correlates with functional recovery.

In the present study we assessed, with sequential retrograde neuronal tracing, the accuracy of tibial motor regeneration after the repair of rat sciatic nerve gaps, either by nerve autografting or by tubulation with a biodegradable chitosan/poly(glycolide-co-lactide) (PGLA) nerve guidance conduit^16^. We also evaluated the function recovery of the hind limb behaviorally and electrophysiologically. Our results showed that tubulation repair of nerve injury both mitigated misdirection of regenerating motor axons and ameliorated chronic contracture in rats.

## Results

### Tubulation repair of the sciatic nerve mitigates misdirection of regenerating tibial motor axons

**Figure 1:**
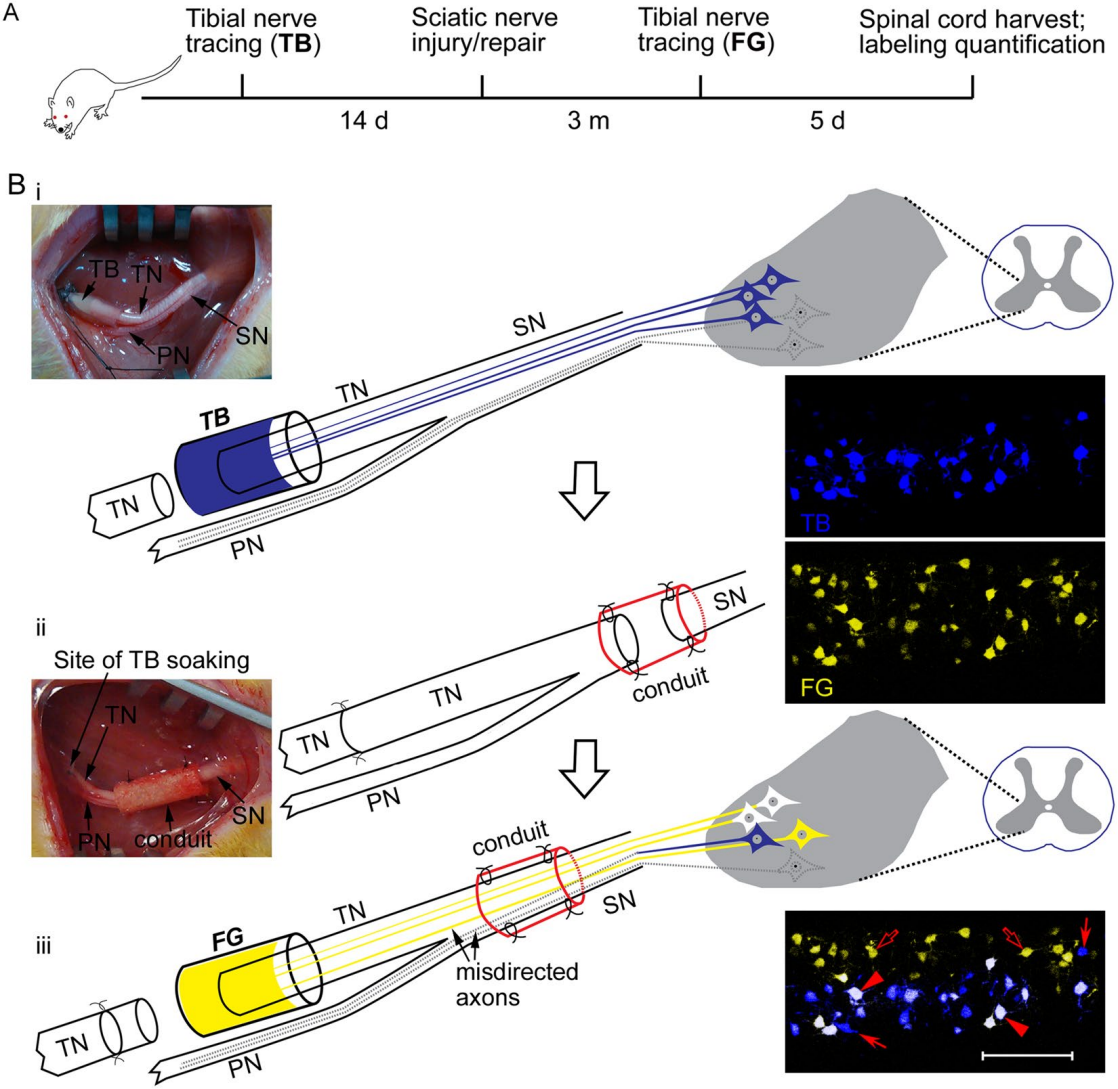
Experimental paradigm (**A**) and schematic diagrams (**B**) showing sequential retrograde neuronal tracing. Sprague-Dawley rats, 3.5 months of age, were subjected to the first retrograde tracing by soaking the proximal stump of the lacerated left tibial nerve into 4% True Blue (TB) for 60 min to label the tibial motor neuron pool in the spinal cord (i). The tibial nerve stump was sutured to the distal one after application of the tracer. Fourteen days later, the left sciatic nerve was subjected to gap injury and subsequent repair with either a nerve autograft or an artificial nerve guidance conduit (ii). Sciatic nerve crush was performed as an additional control. Three months after the sciatic nerve injury and repair, the left tibial nerve was lacerated again but at 2 mm proximal to the previous laceration site, and the proximal stump of the tibial nerve was submerged into 5% FluoroGold (FG) for 60 min to label the spinal motor neurons that had regrew axons into the tibial nerve (iii). Five days later, rats were sacrificed for confocal microscopy to assess regeneration accuracy. The tibial motor neurons which faithfully re-innervated the tibial nerve were labeled by both TB and FG, showing white color (arrowheads), whereas those which failed to re-innervate the tibial nerve or did not regenerate axons at all were labeled by TB alone (filled arrows). The peroneal motor neurons whose axons were misdirected into the tibial nerve were labeled by FG alone (open arrows). *SN*, sciatic nerve; *TN*, tibial nerve; *PN*, peroneal nerve.

**Figure 2:**
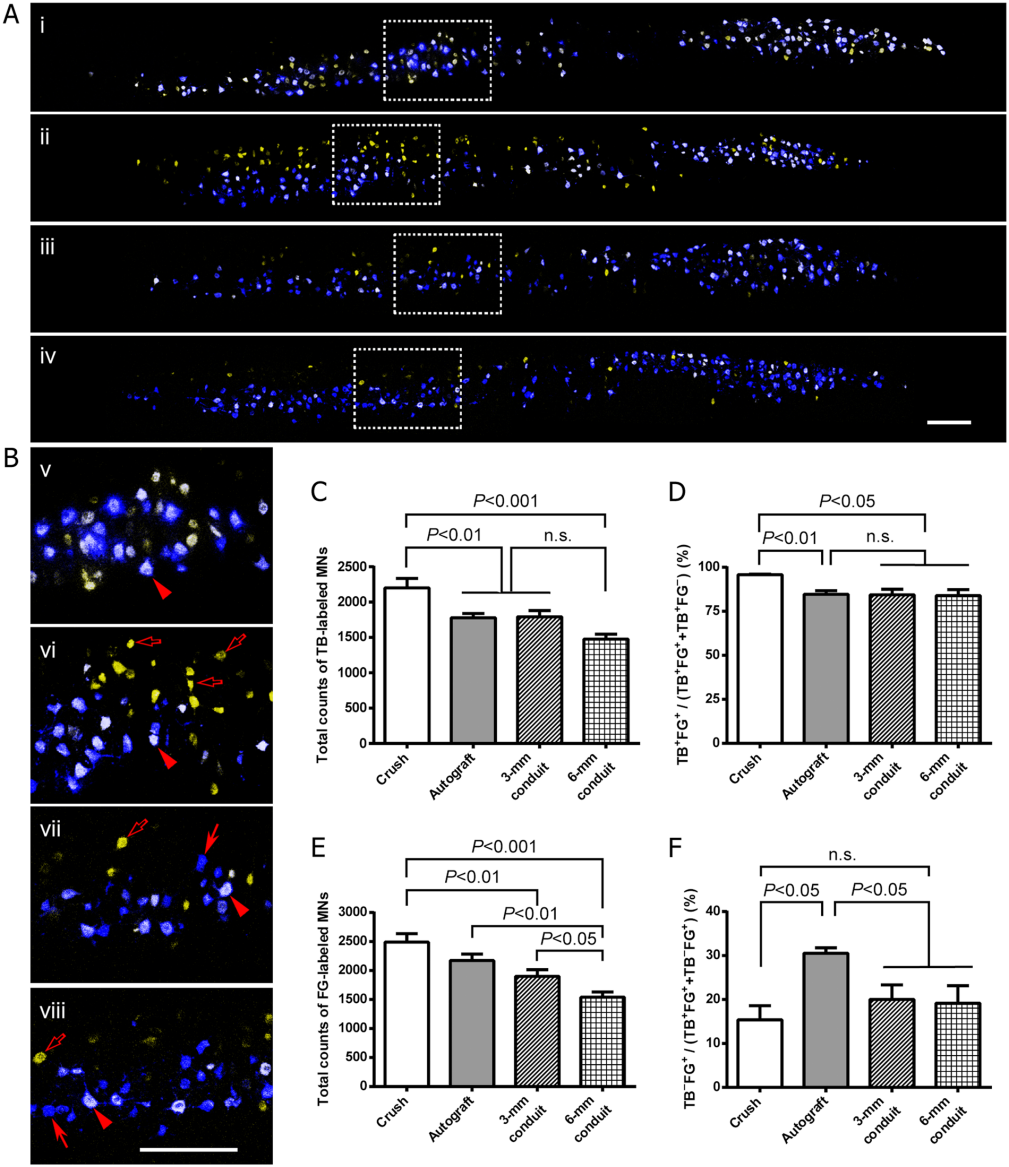
Tubulation repair of the sciatic nerve gap mitigates misdirection of regenerating motor axons into the tibial nerve. (**A**) Confocal photomicrographs showing retrogradely labeled motor neurons within the ventral horn of the spinal cord 3 months after nerve repair. Representative images for the sciatic nerve crush (i), autograft (ii), 3-mm conduit (iii) and 6-mm conduit (iv) groups were shown. (**B**) Magnified view of labeled motor neurons. Sub-panels (v–viii) showed the magnification of the boxed areas in (i–iv), respectively. (**C**–**F**) Quantification of labeled motor neurons. The nerve crush group showed a significantly larger total number of TB-labeled motor neurons (**C**) and higher tibial-to-tibial regeneration ratio, i.e. percentage of TB-FG double-labeled neurons over all TB-labeled neurons (**D**), than any other groups. Notably, the tubulation repair groups showed mildly but significantly reduced number of motor neurons which regenerated and re-innervated the tibial nerve as the gap length increased from 3 mm to 6 mm (**E**); the misdirection of the peroneal motor axons into the tibial nerve was significantly reduced in both 3-mm and 6-mm conduit repair groups as compared to that in nerve autograft group (**F**). Data are expressed as mean±SEM (n = 6 rats each). Scale bar = 300 μm.

To evaluate the effect of tubulation repair on accuracy of motor nerve regeneration, we employed True Blue (TB) to retrogradely label the tibial motor neuron pool 2 weeks prior to sciatic nerve injury, and labeled the motor neurons which had regenerated their axons into the tibial nerve with a second neuronal tracer FluoroGold (FG) at 3 months after sciatic nerve repair (Fig. 1). Confocal photomicrographs of horizontal sections of the spinal cord showed easily distinguishable somata of motor neurons within the ventral horn of the lumbar spinal cord (Fig. 2A,B). The nerve autograft group showed more neurons that were labeled by FG alone when compared to other groups (Fig. 2B).

Quantitation data showed that the total number of TB labeled motor neurons, which represented tibial motor neuron pool, moderately decreased in nerve autograft and 3- and 6-mm conduit groups as compared to sciatic nerve crush (Fig. 2C). Secondly, the tibial motor neurons which faithfully regenerated axons into the tibial nerve, thus dual-labeled by both TB and FG, exhibited a significantly lower ratio of TB^+^FG^+^/TB^+^ in all nerve gap/repair groups compared to nerve crush group (Fig. 2D). Thirdly, the total number of FG-labeled motor neurons, i.e. motor neurons regenerating their axons into the tibial nerve, showed a lower level in 6-mm conduit group as compared to any other groups; no significant difference in the number of FG-labeled motor neurons was seen between nerve autograft and 3-mm conduit groups (Fig. 2E). Finally but intriguingly, in nerve autograft group ~30% of motor neurons whose axons re-innervated the tibial nerve belonged to non-tibial motor neurons (labeled by FG alone); however, in 3- and 6-mm conduit groups the misdirection ratio, TB^−^FG^+^/FG^+^, was reduced by ~33% as compared to that in the nerve autograft group (Fig. 2F).

### Tubulation nerve repair ameliorates chronic flexion contracture

**Figure 3:**
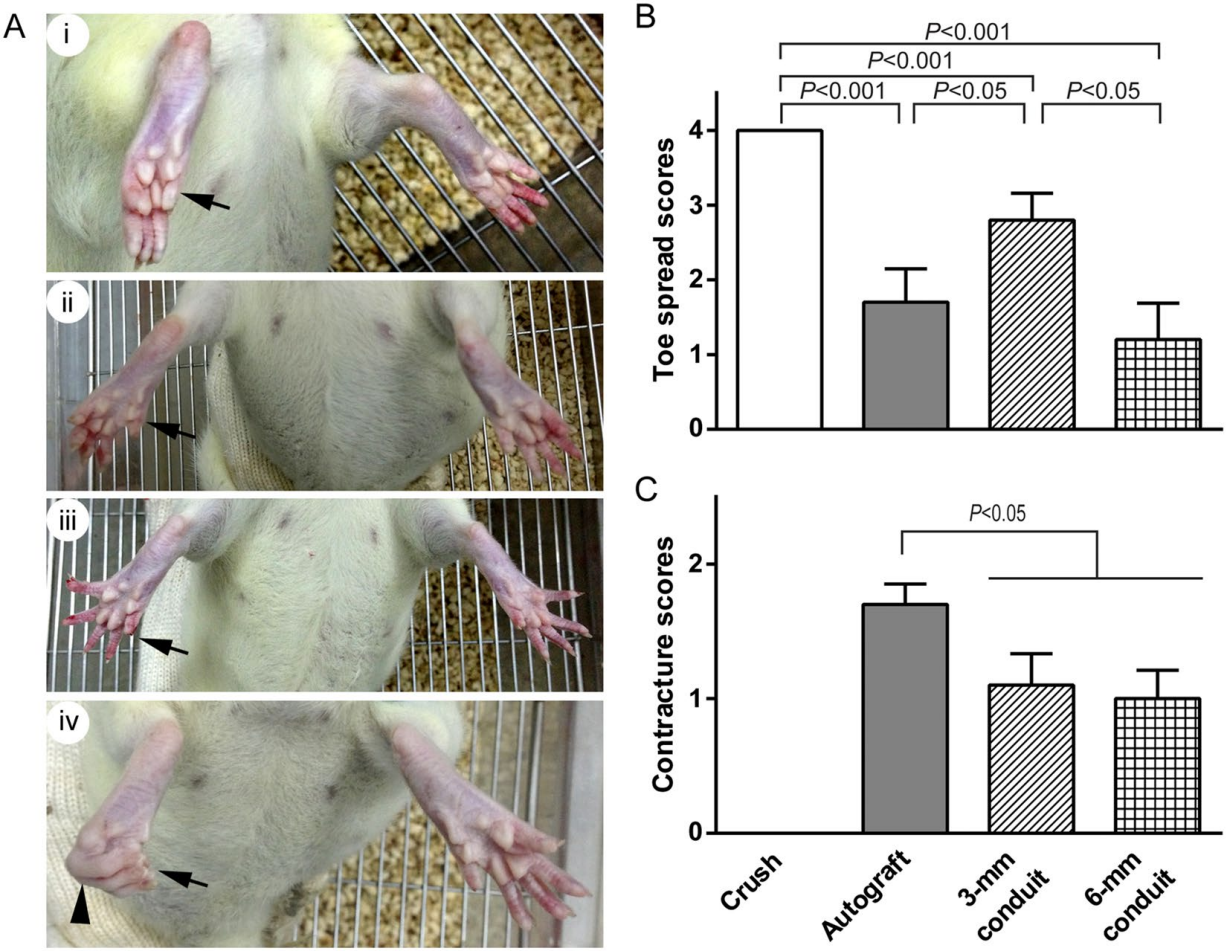
Tubulation repair of the sciatic nerve gap ameliorates chronic flexion contracture of the paw. (**A**) Representative images of toe spread showing different stages of functional motor recovery of the foot. Rats were suspended by the tail and the number of toes showing visible abduction was counted when the rat was trying to escape and its toes maximally spread. At the denervation stage or in the case of no re-innervation, no signs of toe spread were observed (i). Partial (ii) and, in certain conditions, complete (iii) recovery of toe spread function were observed after successful muscle re-innervation. In some cases, the re-innervated foot showed flexion contracture of the toes (arrowhead) with or without toe loss (iv). Arrows indicate the injured foot. (**B**) Bar chart showing toe spread scores at 3 months post-injury. Despite that all nerve gap/repair groups showed incomplete recovery of toe spreading, the 3-mm conduit group exhibited significantly improved toe spread scores as compared to nerve autograft. (**C**) Severity of chronic flexion contracture at 6 months post-injury. The nerve autograft group showed severe flexion contracture; however, the nerve conduit groups showed significantly less severity in contracture. No contracture was observed after nerve crush injury. Data are expressed as mean±SEM (n = 10 rats each).

To examine the effect of tubulation repair on functional recovery after sciatic nerve injury, we performed toe spread test at 3 and 6 months after nerve repair (Fig. 3A). Although none of the nerve gap/repair groups recovered toe spread to the normal level as seen in nerve crush group at 3 months post-injury, the 3-mm conduit group showed an increase in toe spread function as compared to autograft and 6-mm conduit groups (Fig. 3B). At 6 months after injury, all rats in the nerve autograft group exhibited flexion contracture of the paw; however, the rats in 3- and 6-mm conduit repair groups showed significantly less severity in contracture (Fig. 3C).

### Tubulation nerve repair leads to comparable muscle re-innervation

**Figure 4:**
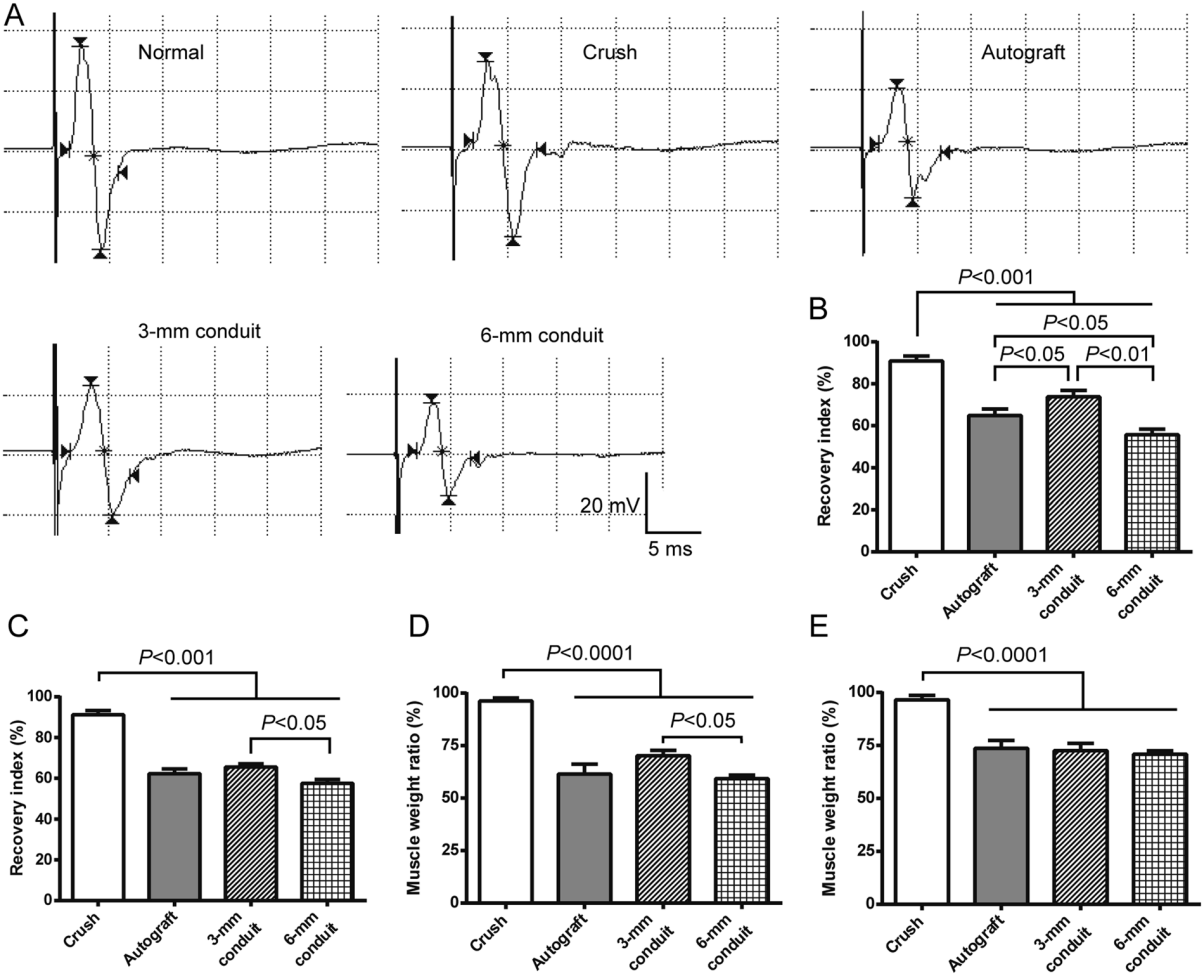
Tubulation repair of the sciatic nerve gap exhibited comparable muscle re-innervation to nerve autografting 6 months post-injury. (**A**) Representative traces of compound muscle action potentials (CMAP) recorded in tibialis cranialis when stimulating the proximal sciatic nerve. The insert shows the scale bar for all traces. (**B**,**C**) Bar charts showing recovery index of CMAP amplitude in tibialis cranialis and gastrocnemius muscles, respectively. Recovery index was calculated as a ratio of peak CMAP amplitude on the injured side over that on the contralateral normal side. The 3-mm conduit group showed significantly improved recovery index compared to nerve autograft group. (**D**,**E**) Wet weight ratio of tibialis cranialis and gastrocnemius muscles, respectively. Data are expressed as mean±SEM (n = 10 rats each).

To observe the effect of tubulation repair of the sciatic nerve on muscle re-innervation, we employed electrophysiological assessment and muscle weight ratio, two sensitive measures of muscle re-innervation^17,18^, at 6 months after nerve repair. Evaluation was carried out in both gastrocnemius and tibialis cranialis, two muscles which are innervated by the tibial and peroneal nerves, respectively. The amplitude of compound muscle action potentials (CMAPs) recorded in tibialis cranialis showed a higher level of recovery in 3-mm conduit group compared to nerve autograft group (Fig. 4A,B); in gastrocnemius muscle, however, the 3-mm conduit group did not differ significantly from nerve autograft group in CMAP amplitude (Fig. 4C). In both muscles, the 6-mm conduit group showed significantly less recovery in CMAP amplitude than the 3-mm conduit group (Fig. 4B,C). None of the three gap/nerve repair groups differed from each other in wet weight ratio of muscles except for a lower weight ratio of tibialis cranialis in 6-mm than in 3-mm conduit groups (Fig. 4D,E).

## Discussion

Misdirection of regenerating axons to inappropriate targets is a strong influential factor that limits functional recovery following peripheral nerve laceration injury and surgical repair^1^, and mitigation of axonal misrouting during regeneration probably represents one of the most challenging tasks in the field of peripheral nerve repair. There is no therapeutic procedure so far to ensure accurate axon-to-axon or endoneurium-to-endoneurium reconnection, and mismatch is unavoidable after nerve anastomosis via epineurial or perineurial suturing, no matter whether direct end-to-end suturing or nerve autografting is executed. In the present study we observed mitigated misdirection of regenerating motor axons and ameliorated chronic flexion contracture by tubulation repair of the lacerated sciatic nerve with a 3- to 6-mm-long bioabsorbable guidance conduit in rats when compared to nerve autografting. To the best of our knowledge, this is the first direct evidence that nerve guides mitigate axonal misdirection and facilitate functional recovery.

Biomaterial nerve guidance conduits, with or without reinforcement by axonal growth-supporting cells, neurotrophic factors and/or luminal biomaterial fillers, have emerged as a promising substitute for nerve autografts to bridge and repair peripheral nerve defects^19–21^. Nerve guidance conduits can restrain trophic molecules released from the distal nerve stump^22^ and facilitate ingrowth of Schwann cells^23^ and thereby support the regrowth of axons from the proximal nerve stump toward the endoneuroal sheaths within the distal stump, along which the regenerating axons finally reach and reinnervate motor or sensory targets^21^. Studies have suggested that motor axons could preferentially reinnervate motor pathways^9^ and axons tend to project to neural target of larger size^24^. However, when the proximal and distal endoneuria tubes are mis-aligned, as in the case of nerve autografting and direct epineurial nerve suturing, the chance of regenerating axons to meet the correct targets is reduced. However, unlike nerve autografting and direct nerve anastomosis in which forced mismatch of endoneuria of different fascicles or different types of axons is unavoidable, tubulation repair of the nerve gap allows for free axonal pathfinding when the regenerating axons reach the interface at the distal nerve stump, and the probability of off-target reinnervation of the distal endoneurial tubes is thus reduced. This could be the reason for mitigated misdirection of regenerating tibial motor axons and ameliorated chronic flexion contracture observed in the present study. This is in line with previous reports that the repair of nerve injury with nerve guidance conduits, intentionally leaving a 2–5 mm gap in between the nerve stumps, improves nerve function recovery in humans^11,12,15^.

The decrease in total counts of TB labeled motor neurons in nerve autograft and 3- and 6-mm conduit groups, as compared to sciatic nerve crush, suggests loss of tibial motor neurons after laceration injury to the sciatic nerve, probably due to combined effect of axotomy and tracer toxicity. We have recently shown that intra-neural injection of neuronal tracers, including FG and TB, results in degeneration of the nerve tissue distal to injection and impairs nerve function^25^; in the present study however, tracer toxicity was balanced across groups since all the rats of the tracing cohort were subjected to prior labeling of the tibial motor neuron pool with TB. Axotomy, especially at a higher level as in the case of sciatic nerve avulsion, leads to apoptosis of motor neurons in adult rats^26^. Loss of tibial motor neurons after nerve autografting and tubulation nerve repair may attribute to delayed and incomplete axonal regeneration due to axotomy with disrupted endoneuria^10,27^. This is also the case for the total number of FG-labeled motor neurons, an indicator of actual motor regeneration into the tibial nerve. It seems that total motor regeneration into the tibial nerve decreases with the length of the nerve gap; however, the regeneration supporting capacity of nerve guidance conduits can be augmented by incorporation of neurotrophic factors, Schwann cells, mesenchymal stem cells or alike^19^.

It should be noted that no stereology was employed in the present study; we manually counted the number of tracer-labeled motor neurons in all serial sections instead. As a consequence, the total number of labeled motor neurons could have been overestimated to a certain degree; however, this potential overestimation was balanced across groups, leaving the data comparable between groups. Also should be noted is that the first tracing surgery involving laceration of the tibial nerve could act as a conditioning injury that might exert influence on the regeneration of the subsequently injured sciatic nerve. It has been shown that a conditioning axonal injury facilities nerve regeneration by increasing the speed of axonal outgrowth^10^; however, it is still unclear whether or not a conditioning injury may influence axonal misdirection during nerve regeneration. Because axonal misdirection results from disrupted endoneurial pathways, it occurs at the interface where two nerve stumps are anastomosed. Given that the sciatic nerve is far proximal to the laceration site of the tibial nerve and that the endoneurial pathways within the sciatic nerve remained intact by the time it was injured, we would not expect substantial influence exerted by the prior tibial nerve injury on misdirection of the sciatic nerve axons. The ~95% regeneration accuracy observed in sciatic nerve crush group in the present study also suggests minimal influence of the prior tibial nerve injury on the accuracy of sciatic nerve regeneration. Importantly, in the present study the potential influence was balanced between groups since all groups were subjected to the same tracing procedures.

To summarize, we found in the present study that tubulation repair of nerve gaps by using a biodegradable chitosan/PGLA nerve guidance conduit both mitigated misdirection of regenerating motor axons and ameliorate chronic flexion contracture of the paw in rats. These results suggest that tubulation repair of a nerve laceration injury by utilizing a bioresorbable nerve guidance conduit represents a potential substitute for end-to-end epineurial suturing and nerve autografting.

## Materials and Methods

### Nerve guidance conduits

Chitosan/PGLA nerve guidance conduits, which comprise an outer tube of chitosan and luminal fillers of PGLA filaments, were prepared as previously described^28^ except that tube-filling filaments were made up of PGLA instead of polyglycolic acid for better biodegradation property. The chitosan tube was 1.2 mm in inner diameter, 0.6 mm in wall thickness, and 5 or 8 mm in length with 3- or 6-mm-long PGLA filaments within the tube lumen, respectively.

### Animals

Sixty-four adult female Sprague-Dawley rats at 3 months of age were used in this study, and all animal procedures were carried out under the approval of Ethics Committee for Laboratory Animals at Nantong University and in accordance with US National Institutes of Health Guide for the Care and Use of Laboratory Animals published by the US National Academy of Sciences. Animals were randomized into 4 groups (16 rats each) and further allocated into 2 cohorts, one for neuronal tracing (n = 6/group) and the other for functional evaluation (n = 10/group).

### Nerve surgery and retrograde neuronal tracing

For the neuronal tracing cohort, sequential retrograde neuronal tracing with TB and FG was employed to evaluate regeneration accuracy (Fig. 1A,B). Based on our previous studies on efficacy and neurodegeneration-inducing action of neuronal tracers^25,29^, TB was chosen as the primary tracer and FG as the secondary one. Fourte**e**n days prior to sciatic nerve injury and repair, rats were subjected to tibial nerve tracing with TB so as to pre-label the tibial motor neuron pool. Rats were deeply anesthetized by intraperitoneal injection of a cocktail anesthetic solution (0.886% w/v sodium pentobarbital, 4.25% w/v chloral hydrate, 2.12% w/v magnesium sulfate, 14.25% v/v ethanol, 33.8% v/v propylene glycol) at a dosage of 2.5 ml/kg body weight. The left tibial nerve was exposed under aseptic condition and transversely cut at 6 mm distal to the bifurcation of the sciatic nerve. The proximal stump of the lacerated tibial nerve was soaked into 5 μl of 4% TB in saline in a custom-made silicone cup. To avoid potential contamination of neighboring nerves and muscles by the tracer, a piece of Parafilm^TM^ at suitable size was placed underneath to isolate the cup and the proximal tibial nerve from surrounding tissues. After soaking in TB for 60 min, the proximal tibial nerve stump was gently cleaned with two changes of saline-presoaked cotton gauze to remove excessive tracer, and then sutured to the distal tibial nerve stump with two stitches of 11/0 nylon sutures in the epineurium. Incisions were closed layer by layer.

Sciatic nerve injury/repair was performed 14 days after primary tibial nerve tracing with TB. Rats were anesthetized, and the sciatic nerve was exposed and injured at 10 mm distal to the sciatic notch. For sciatic nerve crush, the nerve was compressed with a pair of 14-cm hemostat forceps for 30 sec. For nerve tubulation models, the sciatic nerve was transected and both nerve stumps were sutured into a chitosan/PGLA conduit with 9/0 nylon sutures, leaving 1 mm of each of the two nerve stumps within the tube lumen. For the 3 mm conduit group, a 5-mm-long conduit was utilized and the gap length between the two nerve stumps was 3 mm. Similarly, for the 6 mm conduit group, an 8-mm-long conduit was used and the nerve gap within the conduit was 6 mm in length. For nerve autograft group, a 6-mm long sciatic nerve was excised and anastomosed back *in situ* via epineurial suturing with 11/0 nylon sutures. The skin incisions were closed and rats were returned to their home cages after completely recovered from anesthesia on a soft heating pad. The surgery was performed by an experimenter who is well trained and experienced in animal surgery.

Three months after sciatic nerve injury/repair, rats were deeply anesthetized and subjected to the secondary tibial nerve tracing. The tibial nerve was transected at 2 mm proximal to the sutures at which the first tracing was performed, and the proximal tibial nerve stump was soaked into 5% FG in saline for 60 min with Parafilm^TM^ protection and the excessive tracer removed as described above. The repaired sciatic nerve was left intact during tracing surgery and the two stumps of the lacerated tibial nerve were sutured. Rats were survived for 5 additional days to allow for maximal retrograde transport of the tracer FG to the perikaya of spinal motor neurons, and sacrificed under deep anesthesia by transcardial perfusion with saline and subsequently buffered 4% paraformaldehyde. The lumbar enlargement of the spinal cord was carefully dissected out, post-fixed overnight in the same fixative at 4 °C, and dehydrated with increasing concentrations, namely 10%, 20% and 30%, of buffered sucrose solution before cryo-sectioned.

Sciatic nerve surgery for the functional evaluation cohort was performed using the same protocol as above stated except that no neuronal tracing was executed, and the rats were sacrificed 6 months post-injury by transcardial perfusion with saline followed by buffered 4% paraformaldehyde.

### Quantification of labeled motor neurons

The dehydrated spinal cord was horizontally (longitudinally) cut into 30-μm-thick serial sections on a cryostat. The serial sections were sequentially mounted on microscopic slides and the motor neurons labeled by TB and/or FG were visualized and all the sections which showed tracer labeling were photomicrographed with an SP2 laser scanning confocal microscope (Leica Microsystems GmbH, Heidelberg, Germany). Both fluorescent tracers were excited by a 365 nm laser but sequentially detected in different channels with distinct ranges of wave length, 410–450 nm for TB and 550–650 nm for FG. The 10× objective lens was used to acquire images, and panorama view of each section was obtained by stitching images in Adobe Photoshop 6.0 software; the neurons which were co-labeled by both TB and FG and by TB or FG alone were manually counted in individual sections by an experimenter who was blind to the identity of groups.

TB-labeled motor neurons represent the tibial motor neuron pool, whereas those labeled by FG are motor neurons which re-innervated the tibial nerve. Therefore, double labeled motor neurons faithfully re-innervated the tibial nerve, whereas the neurons labeled by TB alone either re-innervated elsewhere or did not regenerate at all; by contrast, the neurons labeled by FG alone represented peroneal motor neurons which misrouted their axons into the tibial nerve during axonal regeneration (Fig. 1). Thus, the ratio of double-labeled neurons to all TB-labeled ones indicated the extent of faithful regeneration of the tibial motor axons that re-innervate the tibial nerve, whereas the ratio of neurons labeled by FG alone to all FG-labeled neurons represents the extent of misdirected peroneal motor axons into the tibial nerve.

### Toe spread test

At 3 and 6 months post-injury, motor recovery of the functional assessment cohort were evaluated with toe spread test as previously described^30,31^ with modification. Briefly, the rat was suspended by the tail, and the maximum of toe spread in the injured hind limb was assessed when the rat was trying to escape, by counting the number of toes with visible abduction and separation from adjacent toes. A 5-tier scoring paradigm was used to assess toe spread function: score 0−no visible toe spread; score 1−one toe spread out; score 2−two toes spread out; score 3−all four toes separated but to a less extent than contralateral normal side; score 4−normal toe spread. In the case that the assessment was impossible, e.g. when severe flexion contracture of the toes was present, a score of 0 was assigned.

The severity of contracture in the toes was assessed with a 3-tier scoring paradigm: score 0−no contracture; score 1−mild contracture with visible toe spread; score 2−severe contracture without detectable toe spread.

### Electrophysiological examinations

Six months after injury, rats of the functional assessment cohort were subjected to electrophysiological recording of CMAPs to evaluate motor nerve conduction and re-establishment of neuromuscular junctions^32,33^. A MYTO portable digital electromyograph recorder (EBNeuro, Italy) was employed for CMAP recording. Briefly, the sciatic nerve proximal to the repair site was re-exposed and excited with maximal electric stimuli by using a bipolar hook electrode, and CMAPs were sequentially recorded in tibialis cranialis and gastrocnemius muscles. The unipolar recording electrode and reference electrode were inserted into the muscle belly and the tendon, respectively. CMAPs of the contralateral normal side were also recorded for calculating the recovery index of CMAP amplitude, i.e. the ratio of peak amplitude of the injured side to that of the contralateral normal side.

### Muscle weight ratio

Six months after injury, rats of the functional assessment cohort were sacrificed, and the gastrocnemius and tibialis cranialis muscles of both sides were carefully dissected out and weighed. The muscle weight ratio, i.e. wet weight of the injured side over that of the contralateral control side, was calculated and compared between groups.

### Statistical analysis

All quantitative data were subjected to one-way analysis of variance (ANOVA) followed by Bonferroni’s *post hoc* comparisons between groups and plotted in the GraphPad Prism 5 sofware package. *P* < 0.05 was considered statistically significant.
